# Juvenile systemic sclerosis: review of 15 patients

**DOI:** 10.1186/1546-0096-12-S1-P118

**Published:** 2014-09-17

**Authors:** Sandra I Sousa, Susana Fernandes, Paula Estanqueiro, Carla Zilhão, Catarina Resende, Filipa Ramos, Manuel Salgado, Margarida Guedes, José Melo Gomes, Maria José Santos

**Affiliations:** 1 Rheumatology, Hospital Garcia de Orta, Almada, Portugal; 2 Rheumatology, Instituto Português de Reumatologia, Lisboa, Portugal; 3 Pediatrics, Hospital Pediátrico de Coimbra; , Coimbra, Portugal; 4 Pediatrics, Hospital de Santo António, Porto, Portugal; 5 Rheumatology, Hospital de Santa Maria, Lisboa, Portugal

## Introduction

Introduction: Systemic sclerosis, a rare disease in childhood, is characterized by skin fibrosis, internal organ involvement, and vasculopathy. Juvenile systemic sclerosis (JSSc) represents less than 10% of all scleroderma patients.

## Objectives

Objectives: To describe the clinical characteristics and disease progression of children with JSSc followed in Portuguese pediatric rheumatology centers.

## Methods

Methods: Clinical and laboratory features as well as medication and outcome of children who met classification criteria for JSSc were reviewed.

## Results

Results: Fifteen patients were identified and included in the analysis, 3 of them were overlap syndromes. Eleven girls (73%), 13 (87%) Caucasians, with a mean age at diagnosis of 11.1±3.0 (3–15) years and a mean disease duration of 7.2±4.2 years (8 months-17 years). In 14 (93%) cases, the first symptom attributable to JSSc was Raynaud's phenomenon, followed by arthritis and/or puffy hands (9 patients, 60%). At disease diagnosis 12 (80%) patients presented periungual capillaropathy and in 8 patients, pulmonary involvement was documented, despite the absence of respiratory complaints. Cumulative disease manifestations as well as complications developed during follow-up are shown in table 1.

**Table 1:** Cumulative manifestations and complications observed during the follow-up

Diffuse cutaneous disease	Digital ulcers	Calcinosis	Musculoskeletal involvement	Interstitial lung disease	Cardiac disease	Gastrointestinal disease	Renal disease
15 (100%)	11 (73%)	3 (20%)	13 (87%)	8 (53%)	2 (13%)	7 (47%)	1 (7%)

All but one child were ANA positive (93%), 7 tested positive for anti-Scl70, 2 positive for anti-RNP and 1 for anti-fibrillarin antibodies. There were no cases of anti-centromere antibodies.

Immunosuppressants (93%), proton pump inhibitors (80%), calcium channel blockers (53%) and corticosteroids (60%) were the most common therapeutic options. Five and four children were treated with prostacyclin analogues and ET-1 receptor antagonist, respectively. One child needed autologous bone marrow transplant due to severe refractory disease.

An improvement of skin thickening and stabilization of pulmonary involvement was documented in most cases. No deaths were registered in this cohort. Table 1.

## Conclusion

Conclusions: Diffuse cutaneous disease was the subtype of JSSc more prevalent identified in pediatric rheumatology centers. Raynaud's phenomenon as well as capillaroscopic abnormalities are almost universal at disease presentation. Internal organ involvement is common and occurs early during disease course, although clinically silent in several cases.

## Disclosure of interest

None declared.

